# Scientific collaboration between ‘old’ and ‘new’ member states: Did joining the European Union make a difference?

**DOI:** 10.1007/s11192-015-1824-y

**Published:** 2015-12-30

**Authors:** Teemu Makkonen, Timo Mitze

**Affiliations:** School of Hospitality and Tourism Management, University of Surrey, Guildford, GU2 7XH UK; Department of Business and Economics, University of Southern Denmark, 6400 Sønderborg, Denmark

**Keywords:** Co-publications, Difference-in-Difference, European Research Area, European Union, Science and technology policy, Scientific collaboration, 91Cxx, I230, O520

## Abstract

This paper provides new insights on the effects of the enlargement of the European Union (EU) and European integration by investigating the issue of scientific collaboration within the new EU member states *vis*-*à*-*vis* the old EU member states. The question addressed is whether the EU membership following the two enlargement waves 2004 and 2007 has significantly increased the co-publication intensity of the new member states with other member countries. The empirical results based on data collected from the Web of Science database and Difference-in-Difference estimations point towards a conclusion that joining the EU indeed has had an additional positive impact on the co-publication intensity between the new and old member states and, in particular, within the new member states themselves. These results give tentative support for the successfulness of the EU’s science policies in achieving a common ‘internal market’ in research. We also find evidence for early anticipation effects of the consecutive EU accession.

## Introduction

The importance of achieving highly collaborative (regional, national or continental) science systems has been discussed in academic literature for decades (Hicks and Katz 1996). While admitting that it might be too straight assumption to consider that the outcomes of collaboration are always positive, it certainly seems that today the issue is increasingly topical in the context of the European Union (EU). The EU is aiming to develop its scientific and research system towards a high degree of integration and collaboration between the different EU member states. However, the continuous expansion of the EU sets obstacles and challenges to this integration process. Evidently there are huge differences in the scientific collaboration patterns between the established EU countries (EU-15^1^) and the new member states (NMS-12^2^) of Eastern and Southern Europe. It is suggestive to think that these differences between the new and old member states would converge as time passes by, however no statistical evidence conclusively verifying this kind of development exists: the impact of the EU membership in the scientific collaboration patterns of the NMS-12 is an issue rarely discussed in the academic literature (Mattsson et al. 2008). Rather, the existing empirical literature either assumes that this is the case or shows only partial evidence of homogenization or convergence, whereas countering arguments and serious doubts of the possibilities of achieving an integrated domestic (EU) market for research have also been stated.

The above controversy acts as the motivation behind our empirical approach. We address the discussed knowledge gap by formulating the following two research questions addressed with straightforward statistical tests (Difference-in-Difference estimations) based on data from the Web of Science database:

1. Is there any significant difference in the cross-border co-publication intensity *between the NMS-12 and EU-15* before and after the EU membership *vis*-*à*-*vis* the benchmark of the cross-border co-publication intensity within the EU-15?
2. Is there any significant difference in the cross-border co-publication intensity *within the NMS-12* before and after the EU membership *vis*-*à*-*vis* the benchmark of the cross-border co-publication intensity within the EU-15?

The remainder of this paper is organized as follows. First, a brief review on the relevant EU strategies and policies together with an overview on the existing empirical literature on cross-border scientific collaboration in the EU are presented. Second, the data gathering process and the limitations it entails are discussed followed by the introduction of our empirical approach. Third, the results of this paper are summarized in accordance with the research questions laid out above together with a series of robustness checks. Discussion and concluding remarks will follow.

## Cross-border scientific collaboration in the European Union

### Science and technology policies and strategies of the European Union

Scientific collaboration and knowledge flows are persistent and recurrent themes in EU policy concerns and documentation for a ‘borderless Europe’. In particular, such a borderless Europe is designed in part to enable scientific collaboration, researcher mobility as well as knowledge transfer and flows between EU nations and regions for the benefit of European national and regional innovativeness. This effort towards a greater collaboration and mobility of researchers has been particularly evident in the strategic documents ‘Europe 2020: A European strategy for smart, sustainable and inclusive growth’ (European Commission 2010) and ‘Lisbon strategy for sustainable economic growth and jobs in Europe’ (Commission of the European Communities 2000) of the EU.

Many European science and technology institutions, networks and policies have emerged that provide funding, incentives and means for collaboration in knowledge production and its subsequent exploitation and dissemination (Stein 2004). For example, the launch of the Framework Programmes for Research and Technology Development, in 1984, is an important milestone in this respect (Barré et al. 2013). In its attempts to move towards a more coordinated approach (Stein 2004), in the Lisbon strategy the EU set goals for becoming the world’s leading community in terms of innovation by building this strategy around the concept of the European Research Area (ERA; European Commission 2000; Commission of the European Communities 2007; Council of the European Union 2008). This discussion was centred on two main concerns: (1) the gap that existed between the EU *vis*-*à*-*vis* USA and Japan in terms of EU’s modest innovation expenditure and outputs and (2) the fragmented nature of EU’s research efforts (Breschi and Cusmano 2004; Hervás Soriano and Mulatero 2010). The ERA concept was, thus, designed particularly for strengthening European competitiveness and achieving an ‘internal market’ in research. In other words, it was aimed at mobilizing knowledge, researchers and technology through the restructuring of the European research fabric towards greater internal EU cohesion and integration that would, at the same time, erase duplicative research efforts within the EU (de Bruijn and Lagendijk 2005; Scherngell and Barber 2011). In effect, the ERA can, thus, be considered to represent ‘the entirety of the EU research policy’ (Luukkonen 2014: 33).

Even though evaluations on the successfulness of the Lisbon strategy have concluded only on partial success of the agenda to achieve the envisioned goals (e.g. Copeland and Papadimitriou 2012), the ERA concept embedded in it has redefined the discourse on European science and technology policy (Pereira 2002; Edler et al. 2004). Consequently, the concept has been transformed into the current Europe 2020 strategy, where one of the flagship initiatives has been designated as ‘Innovative Union’ (Hervás Soriano and Mulatero 2010). Within this initiative the EU has set out to enhance cross-border collaboration and to ensure the diffusion of technology across the EU territory. This is envisioned to be partly achieved by ‘completing the ERA’. Therefore, in short, the ethos of the ERA can be summarized through its goals of enabling researchers, research institutions and businesses to increasingly circulate and cooperate across borders.

While non-member states can benefit from the funding and networking opportunities of the EU through varying neighbourhood policies (European Commission 2012), once a country joins the EU it becomes fully a part of the ERA. This should then increase the potential for scientific collaboration with partners from other EU countries. Thus, from a policy perspective it is tempting to believe that the recorded increase in intra-EU networking and the increasing number of member states participating in these networks (as the empirical results discussed below indicate) stem from policy induced collaborations (Mattsson et al. 2008). However, serious doubts can be raised whether the current ERA policies are enough to create cohesive research collaboration with equal possibilities across the whole of the EU, particularly between businesses (Archibugi and Coco 2005; Hoekman et al. 2010). In line, Ponds (2009) has noted that whereas the absolute numbers of international co-publications might have increased, their share of the total publications has remained the same. This observation has led Ponds (2009: 76) to declare that ‘the process of internationalization has reached an end’. Accordingly, thus far no conclusive macro-level evidence has been found to support a notion that a highly interconnected ERA would have been achieved (Tijssen 2008).

### Empirical evidence on the integration of science in the European Union

When looking at the sheer number and variety of EU funded collaborative projects and programmes it seems that an integrated and common European knowledge system appears to be emerging. The coordination of European science and technology policies through the ERA reinforces this notion (Stein 2004). Moreover, earlier empirical evidence has pointed towards the ‘Europeanization’ of shared research and development activities, co-authorship and contacts by academic staff within Europe rather than internationalization outside Europe (Hoekman et al. 2010; Barré et al. 2013). In other words, cross-border research collaboration has become increasingly directed towards other European countries at the expense of inter-continental co-authoring (Smeby and Trondal 2005). Thus, it seems that the aims of achieving an internal market for research have, to some extent, been met (irrespective of whether this is a direct impact of EU strategies and the ERA or a coincidence). Furthermore, several studies have hypothesized on the significance and impact of the EU membership in boosting research collaboration of the NMS-12 with the established EU-15 (Marshakova-Shaikevich 2006, 2007). It indeed seems that: (1) in terms of co-publications the NMS-12 are more EU focused than the EU-15, (2) the intra-EU connectivity of the NMS-12 is currently catching up to the established ones at a rapid pace (Tijssen 2008; Hoekman et al. 2010) and (3) international collaboration among researchers from the Eastern European countries seems to have been boosted recently (Kozak et al. 2015). Contrarily, for example Cecere and Corrocher (2015) have stated that the collaborations between the different tiers of EU members (namely the EU-15 and NMS-12) are still less frequent and weaker than collaborations within the established EU countries.

Therefore, despite some positive signs of the success of the EU’s policy instruments for achieving a common ‘home-market’ for research (Glänzel and Schlemmer 2007; Roediger-Schluga and Barber 2008; Scherngell and Lata 2013), the existing empirical literature points towards a conclusion that scientific collaboration in the EU is still most typically done in alliance with partners from a shared home country (Okubo and Zitt 2004; Puuska et al. 2014), even though the advancements in information and communication technologies have significantly enhanced the ease of ‘being in touch’ with partners over great distances (Gallié and Guichard 2005). Thus, evidently, national borders and geographical distance—in addition to e.g. financial resources available for researchers and their individual motivation (Jeong et al. 2014; Ukrainski et al. 2014), technological (Scherngell and Barber 2009; Barber and Scherngell 2013) and institutional proximity (Ponds et al. 2007; Ponds 2009) together with cultural, historical and language issues (Acosta et al. 2011; Plotnikova and Rake 2014)—still have an impact on the scientific collaboration patterns inside the EU. Indeed, whether measured in patents (Greunz 2003, 2005), publications (Hoekman et al. 2009, 2010), web domains (Ortega and Aguillo 2008) or collaborative projects (Constantelou et al. 2004; Cecere and Corrocher 2015) scientific collaboration and knowledge spillovers still tend to concentrate nationally and regionally and cross-border and inter-regional collaboration is more prominent between countries and regions that are geographically close *vis*-*à*-*vis* distant countries and regions.

In addition, there is also an evident tendency to collaborate with established partners, since long traditions of collaboration and trust play significant roles in the process of selecting partners. Moreover, the ERA has also fostered and reinforced the ‘centralization of knowledge flows among already well-connected excellence and capital regions’ (Hoekman et al. 2009: 736). This ‘path-dependency’ might turn out problematic for the research institution in the NMS-12, which need time to earn the trust of the other actors as well as a tradition of partnership before they can break into the ‘oligarchic’ core networks of scientific collaboration of the EU (Breschi and Cusmano 2004; Must 2010). In fact, whereas for example Finland, who joined the EU in 1995, has been able to integrate well into the ERA (Toivanen and Suominen 2015), serious concerns on the internationalization of science in the post-communist countries were raised already in the early phases of their transition, since the observed ephemeral nature of the international collaboration networks supported only the most advanced scientists and research with a strong similarity to the ‘West’ (Mirskaya 1997). However, despite the ample empirical work on measuring and analyzing the trends in international scientific collaboration, in general, the impact of the EU member state status on the patterns of scientific collaboration and the integration processes in European science remain, in particular, understudied (Mattsson et al. 2008; Ukrainski et al. 2014) and, thus, are under empirical scrutiny here.

## Data and methods

### Data

The data on the co-authored article publications was gathered form the Web of Science (WoS) database (during January 2014). In relation to their reliability as an indicator, it has to be noted that scientific co-authorships of publications do not measure the whole universe of scientific collaboration (Katz and Martin 1997; Laudel 2002). Moreover, the database used here covers only articles published in journals indexed in WoS leaving a multitude of scholarly journals outside the scope of this study. Additionally, there are limitations in the data gathering and processing phases of bibliometric variables, which make co-authored publications an error-prone indicator (Luukkonen et al. 1993). However, (with prober diligence paid on the data collection phase) they are arguably among the best and the most commonly applied indicators of international scientific output and collaboration and WoS among the best sources for this kind of data (Moed et al. 2005; Wagner and Leydesdorff 2005).

**Fig. 1:**
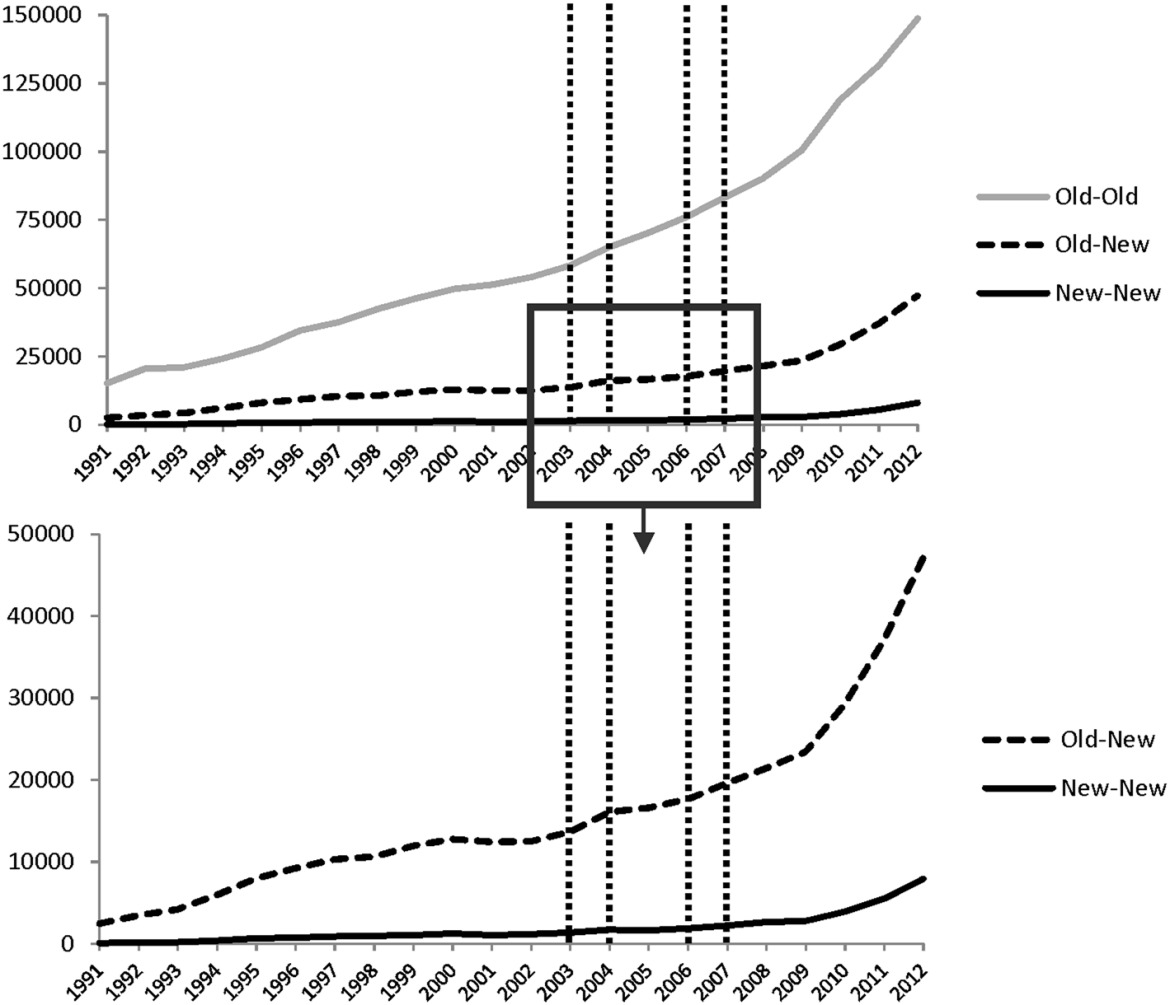
Collaboration counts in the dataset between the old and new EU member states. *Source*: Own calculations based on data from the WoS database

The data was gathered for a time period covering from 1991, signalling independence for many of the NMS-12, to 2012. The first two years of the observation period included information for the Czech Republic and Slovakia even though Slovakia gained its independence only in January of 1993. The latest EU member, namely Croatia, was left outside the analysis, since it joined the EU only in 2013. The collaboration intensities between countries were identified by a search procedure, following the Boolean logic embedded in the WoS database, by including both countries in the search fields of the authors responsible for the publication. Thus, the numbers of joint-publications presented here cannot be considered as the sum total of scientific collaboration, as some of the publications are bound to be counted several times in our data, when the publication has authors from many EU member countries. Similarly, a single-authored journal article by an author possessing an institutional address in two (or more) EU countries is, in fact, counted here as cross-border scientific collaboration. At the same time it has to be acknowledged that an author working jointly in two countries is expected to collaborate to a certain degree with colleagues from both home institutions (Trippl 2013). Still, we do acknowledge that when working with bibliometric data there will always be sources of errors such as similar names, misspellings etc. (Erman and Todorovski 2015). However, when taking into account the whole dataset and our approach on comparing country groups, we consider this ‘noise’ to remain as negligible. In sum, the publication counts are used here to indicate the volume of change rather than show exact numbers of jointly published articles within the EU as a whole (Fig. 1) for: the EU-15 (‘Old’), the NMS-12 (‘New’), the new member states of the 2004 enlargement i.e. NMS-10 as well as Bulgaria and Romania i.e. NMS-2. Additionally the total numbers of publications in WoS by countries were retrieved.

Although some of the growth in co-publications counts can be explained by the growing number of articles covered by WoS (Michels and Schmoch 2012), Fig. 1 already depicts two apparent stylized facts: firstly, the overall level of co-publications is clearly the highest within the group of EU-15 (Old–Old), followed—but with a large intercept—by the level of co-publications between the old and new member states (Old-New), where the latter includes all 12 new members from both enlargement waves 2004 and 2007, as well as within new member states (New–New). Secondly, all three time series grow significantly over the time period considered, particularly starting in the second half of the last decade. From this visual inspection, however, it cannot be inferred with statistical precision if—controlling for the initial level differences in the number of co-publications among the three groups—one of these time trends outperforms the others. Thus, in order to shed more light on this latter issue, we apply a commonly used statistical estimation approach, which allows us to analyze whether the EU membership has led to a statistically significant ‘excess’ growth in the co-publication behaviour between the old and new member states (Old–New) as well as within new member states (New–New) compared to the ‘baseline’ trend in the co-publication behaviour within old member states (Old–Old) or not.

### Methods

The Difference-in-Difference (DiD) approach is a quantitative research design for estimating causal relationships in quasi-experimental settings. It is popular, for example, in empirical economics as well as other social sciences and commonly applied when estimating the effects of certain policy interventions or institutional changes that do not affect everybody at the same time. The great appeal of the DiD-approach is its conceptual rigor and computational simplicity: the approach consists of identifying a specific intervention or treatment (e.g. a change in the political regime, the passage of a law, etc.); and comparing the difference in outcome levels or growth rates before and after the intervention for groups that are affected by the intervention to the same difference for unaffected groups (for the purpose of this study: joining the EU; Bertrand et al. 2004; Lechner 2010). Throughout our empirical identification strategy, the collaboration counts within the EU-15 act as the baseline against which the other treatments are benchmarked. Thus, here we identify the effects of accession of new member states by isolating countries that have recently joined the EU and comparing the changes in international (intra-EU) scientific co-publishing with countries already belonging to the EU. Given the distinct nature of our underlying dyadic co-publication data between EU countries, the application of DiD-estimation can be seen as ‘an attractive choice when using research designs based on controlling for confounding variables or when using instrumental variables is deemed unsuitable, and at the same time, pre-treatment information is available’ (Lechner 2010: 167).

The basic setup of DiD-estimation thereby involves the classification of one or more treatment groups, a comparison group as well as the specification of outcome and treatment variables, where the latter divides the time dimension of the analysis into (at least) one pre-treatment and post-treatment period. The idea of the empirical identification strategy of DiD-estimation is then to compare the evolution of the mean value of the outcome variable for the treatment and comparison group over time, where the inclusion of the latter comparison group is essential to account for a common (global) time trend in the outcome variable across groups that is not attributable to the treatment. If the DiD-estimation setup, by means of the definition of the treatment, and comparison group as well as the exact timing of the treatment is properly specified, it can then be seen as a proxy of the essential but unobserved counterfactual question of ‘What would have happened to the mean outcome level of the treatment group if, everything else equal, the group had not been subject to the treatment?’, which is needed to make statements with regard to the ‘causal’ impact of the treatment on the outcome variable.

**Fig. 2:**
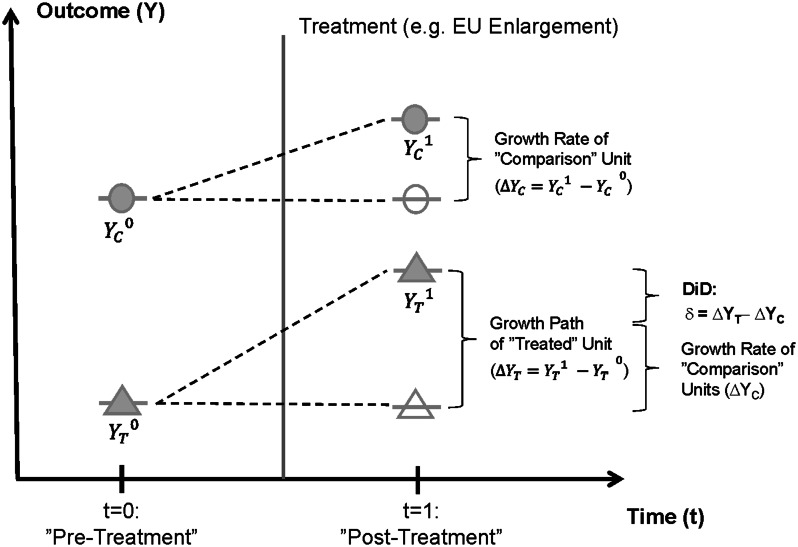
Presentation of the DiD-calculation method for treated and comparison units

Formally, the DiD-estimation approach combines the use of cross-sectional and time series data and aims at measuring the changes in an outcome variable *Y* of treated units (*T*) and comparison units (*C*) before and after a treatment *D* has taken place. Thus, the DiD-approach conducts a joint ‘before–after’ comparison in the change of *Y* for the treatment group over time together with a ‘cross-sectional’ comparison in the levels of the outcome variable for both the pre- and post-treatment time period. The underlying logic of the DiD-approach is graphically shown in Fig. 2. The figure displays the level in the outcome variable *Y* for a treated unit (triangle), both before (*t* = 0) and after treatment (*t* = 1). The associated outcome levels are denoted as $Y_{T}^{0}$ and $Y_{T}^{1}$, respectively. As shown in Fig. 2, the observed level of the outcome variable increases from the pre- to the post-treatment period. The growth rate in a before–after comparison can be written as $\Delta Y_{T} = Y_{T}^{1} - Y_{T}^{0}$.

However, not only the outcome of the treated unit, but also the output level of the comparison unit (circle) is observed *vis*-*à*-*vis* growth over the time period of analysis in the example of Fig. 2 according to $\Delta Y_{C} = Y_{C}^{1} - Y_{C}^{0}$. Moreover, in both periods the level of the outcome variable for the comparison unit is higher than the level of the outcome variable of the treatment unit (in line with the time trends shown in Fig. 1). Thus, ignoring initial level differences and the existence of a common (global) time trend for the outcome variable in focus, which is not attributable to the treatment but equally present for both the treated and comparison unit, might lead to an over-estimation of the causal effect of the treatment on the treated as shown in Fig. 2. The DiD-approach controls for this source of estimation bias by calculating the difference of the two growth rates as $\Delta Y_{T} - \Delta Y_{C}$. The resulting difference in the time differences is the so-called DiD-parameter (*δ*), which can be quantified for a sample of observations in a regression approach.

Statistical inference in a regression framework is typically conducted using group averages of the outcome variable $\left( {\bar{Y}} \right)$ for treated and comparison units. The DiD-parameter for quantifying the average treatment effect of the treated (ATT) can then be written as

1 $$\delta_{ATT} = \left( {\bar{Y}_{T}^{1} - \bar{Y}_{c}^{1} } \right) - \left( {\bar{Y}_{T}^{0} - \bar{Y}_{c}^{0} } \right) = \bar{Y}_{T}^{1} - \bar{Y}_{C}^{1} - \bar{Y}_{T}^{0} + \bar{Y}_{C}^{0} = \Delta \bar{Y}_{T} - \Delta \bar{Y}_{C} ,$$

where the above parameter can be either defined as the ‘before–after difference’ in the ‘cross-group difference’ or the cross-group difference in the before–after differences as summarized in Table 1. One has to note that the validity of the DiD-estimation approach rests on certain assumptions. Firstly, it is assumed that the specified comparison group identifies the common time path of the outcome variable that would have happened in the absence of the treatment. In other words, the common trend assumption states that if the treated had not been subject to the treatment, both groups would have experienced the same time trend (Lechner 2010). Thus, a potential estimation bias in the DiD-approach arises in situations where something else than the treatment changes in one group but not in the other at the same time as the treatment. Secondly, another important assumption of the DiD-approach is bias stability (Lechner 2010). This assumption states that the treatment has no impact on the level of the outcome variable in the pre-treatment outcomes and therefore any observed difference in the pre-treatment period between groups can be used to correct the observed differences in post-treatment outcomes.

**Table 1:** DiD-parameter definition based on sample averages of treated and comparison units

	Before treatment	After treatment	“Before–After” difference
Average Group 1 (treated)	$\bar{Y}_{T}^{0}$	$\bar{Y}_{T}^{1}$	$\Delta \bar{Y}_{T} = \bar{Y}_{T}^{1} - \bar{Y}_{T}^{0}$
Average Group 2 (comparison)	$\bar{Y}_{C}^{0}$	$\bar{Y}_{C}^{1}$	$\Delta \bar{Y}_{C} = \bar{Y}_{C}^{1} - \bar{Y}_{C}^{0}$
“Cross-Group” difference	$\bar{Y}_{T}^{0} - \bar{Y}_{C}^{0}$	$\bar{Y}_{T}^{1} - \bar{Y}_{C}^{1}$	$$\begin{aligned} &\delta_{ATT} = \left( {\bar{Y}_{T}^{1} - \bar{Y}_{C}^{1} } \right) - \left( {\bar{Y}_{T}^{0} - \bar{Y}_{C}^{0} } \right) \hfill \\ &= \bar{Y}_{T}^{1} - \bar{Y}_{C}^{1} - \bar{Y}_{T}^{0} + \bar{Y}_{C}^{0} = \Delta \bar{Y}_{T} - \Delta \bar{Y}_{C} \hfill \\ \end{aligned}$$

In a regression framework, we can obtain empirical estimates for the average treatment effect on the treated (ATT) from a fixed effects model (FEM) for dyadic data, which is able to control for unobserved time-fixed effects among country pairs as

2 $$y_{ij,t} = \mu_{ij} + \sum\limits_{g = 1}^{G} {\delta^{2004,g} \left( {D_{t}^{2004} \times T_{ij}^{g} } \right)} + \sum\limits_{g = 1}^{G} {\delta^{2007,g} \left( {D_{t}^{2007} \times T_{ij}^{g} } \right)} + \varepsilon_{ij,t} ,$$

where the indices *i, j* denotes the cross-sectional dimension of the data with *i, j* = 1,…,*N* (in our case EU-27 countries, where we exclude intra-country combinations by setting *i* *≠* *j*) whereas *t* is the time dimension with *t* = 1,…,*T*. $y_{ij,t}$ denotes the outcome variable of the regression equation, which is defined as the log-transformed share of pairwise co-publications (*pub*) for the country pair *i, j* (with *i, j* = 1,…,27) in the average total number of co-publications for both countries *i, j* as

$$y_{ij,t} = \log \left[ {\frac{{pub_{ij,t} }}{{\left( {\sum\nolimits_{i} {pub_{ij,t} } + \sum\nolimits_{j} {pub_{ij,t} } } \right)/2}}} \right].$$

Our motivation for using the pairwise co-publication intensity for country pair *i, j* in the two countries’ average total number of intra-EU co-publication levels rather than absolute counts stems from theoretical as well statistical considerations. From a theory perspective, using co-publication intensities allows linking our analysis to the argumentation outlined in Ponds (2009) noting that investigating the evolution of shares of international co-publications rather than absolute numbers is a better indicator for assessing whether the process of internationalization has reached an end or not. From a statistical perspective, there are two arguments in favour of the above defined outcome variable. Firstly, using co-publication intensities allows minimizing the risk of running spurious regressions for non-stationary variables as shown in Fig. 1 for co-publication counts (Granger and Newbold 1974). Secondly, comparing different variables such as (1) co-publication counts in levels, (2) log-transformed co-publication counts, (3) co-publication intensity in levels and (4) log-transformed co-publication intensity, only for the latter variable we cannot reject the null hypothesis of a normal distribution, which is needed in order to apply ordinary least squares-based (OLS-based) DiD-estimation. Since the choice of our outcome variable results in a log-level specification of Eq. (2), the obtained regression coefficients can be interpreted in terms of percentage changes for discrete variations in the DiD-terms. Descriptive statistics of the defined co-publication intensity are given in Table 2.

**Table 2:** Summary statistics of co-publication intensity

Variable	Definition	Obs.	Mean	SD	Min	Max
Co-publication intensity (in %)	$\left[ {\frac{{pub_{ij,t} }}{{\left( {\mathop \sum \nolimits_{i} pub_{ij,t} + \mathop \sum \nolimits_{j} pub_{ij,t} } \right)/2}}} \right]$	7722	0.994	1.2404	0.00	10.01

For further details about variable definition, see main text

In Eq. (2), the multiplicative terms $\left( {D_{t}^{2004} \times T_{ij}^{g} } \right)$ and $\left( {D_{t}^{2007} \times T_{ij}^{g} } \right)$ are the crucial variables in the DiD-approach for estimating the co-publication effect of EU enlargement. Thereby, the variables $D_{t}^{2004}$ and $D_{t}^{2007}$ are binary flag indicators, which take values of zero before 2003 and 2007, respectively, and have values of one afterwards as

$$D_{t}^{2004} = \left\{ {\begin{array}{*{20}l} 1 \hfill & {{\text{if}}\;t \ge 2004} \hfill \\ 0 \hfill & {{\text{if}}\;t < 2004} \hfill \\ \end{array} } \right.\quad {\text{and}}\quad D_{t}^{2007} = \left\{ {\begin{array}{*{20}l} 1 \hfill & {{\text{if}}\;t \ge 2007} \hfill \\ 0 \hfill & {{\text{if}}\;t < 2007} \hfill \\ \end{array} } \right..$$

The purpose of these to binary dummy variables is, thus, to indicate the timing of the two EU enlargement waves 2004 and 2007, respectively. The variable $T_{ij}^{g}$ is a group variable, which assigns the *i, j*th country pair to one of the following *g* = 1,…,6 macro groups as $T_{ij}^{1} = 1$ if both countries *i* and *j* are EU-15 member states (and is zero otherwise), $T_{ij}^{2} = 1$ if country *i* is a member of the EU-15 and country *j* is a member of the NMS-10 (vice versa and is zero otherwise), $T_{ij}^{3} = 1$ if country *i* is a member of the EU-15 and country *j* is a member of the NMS-2 (vice versa and is zero otherwise); $T_{ij}^{4} = 1$ if country *i* is a member of the NMS-10 and country *j* is a member of the NMS-2 (vice versa and is zero otherwise), $T_{ij}^{5} = 1$ if both countries *i* and *j* are NMS-10 member states (and is zero otherwise) and finally $T_{ij}^{6} = 1$ if both countries *i* and *j* are NMS-2 member states (and is zero otherwise).

The multiplicative interaction terms constituting of these six group dummies together with the two specified time dummies then allow quantifying the group-specific time trends measuring the change in the co-publication intensity in the course of the EU accession (that is, the before–after difference according to Table 1). The parameters $\sum\nolimits_{g = 1}^{G} {\delta^{2004,g} }$ thereby measure the overall growth rate for each group before and after 2004 (up to 2012), while the parameters $\sum\nolimits_{g = 1}^{G} {\delta^{2007,g} }$ estimate any additional growth effect in the period after 2007 (up to 2012). In the course of estimation, the first group $(T_{ij}^{1} )$ will be used as the baseline growth scenario. We use a series of nonlinear tests for the combination of estimates based on the delta method in order to assess the null hypothesis of equal growth rates between the five treatment groups and the comparison group $(T_{ij}^{1} )$ as

3 $$H_{0} : \left( {\delta^{2004,g} + \delta^{2007,g} } \right) - \left( {\delta^{2004,1} + \delta^{2007,1} } \right) = 0\quad \left( {for\;g = 2, \ldots ,6} \right).$$

If the tests reject the validity of the null hypothesis for some of the groups against the alternative hypothesis that the difference is larger than zero, then we observe a statistically significant EU membership effect based on our underlying dyadic co-publication intensity (or in other words: a statistically significant excess growth for some of the treatment groups). Thus, the long-run average treatment effect of the treated for each of the five treatment groups can be defined as

4 $$\delta_{ATT}^{g} = \left( {\delta^{2004,g} + \delta^{2007,g} } \right) - \left( {\delta^{2004,1} + \delta^{2007,1} } \right)\quad \left( {for\;g = 2, \ldots ,6} \right).$$

The reader has to note that we apply a symmetric setup up for the estimation of the long-run average treatment effects. That is, we allow EU enlargement effect for the NMS-10 also to be present throughout the time period of the second enlargement wave after 2006. Our motivation for doing so is that, on the one hand, the adoption of new co-publication strategies involving the new member states in the course of EU enlargement may take some time and only gradually adjusts with a time lag. On the other hand, the late but pre-known access of Bulgaria and Romania (NMS-2) may have triggered early anticipation behaviour in co-publication intensities, which may already be visible in the course of the first wave of EU enlargement 2004. We will put an explicit focus on the role of early anticipation of EU enlargement in “Robustness checks” section by running a series of robustness checks to our overall regression approach.

Another form of symmetry applies to our general data setup. That is, given that we have undirected dyadic data at hand; the relationship $y_{ij,t} = y_{ji,t}$ holds. Excluding intra-country co-publications (*i* *≠* *j*), this gives us a total number of *N*·(*N* − 1) = 27·26 = 702 observations. The total number of observations for all 22 sample year is *N*·(*N* − 1)·*T* = 15,444. However, if we do not control for the symmetric information in the dyadic data setup, we would get an over-precision in the estimation results, which would make the regression output highly unreliable. Thus, we solve this problem by deleting all double information leaving total number of observations for estimation as [*N*·(*N* − 1)]/2·*T* = 7,722. A further important element in the regression framework of Eq. (2) is the inclusion of country-pair fixed effects $(\mu_{ij} )$, which capture the non-random nature of the treatment by means of EU membership to countries with systematically lower average levels of the outcome variable $(\bar{y}_{ij,t} )$. Finally, $\varepsilon_{ij,t}$ is a standard *i.i.d.* error term. In a panel data setup, the inclusion of country-pair fixed effects also implies that no treatment group dummies need to be added to the regression frameworks since all-time invariant variables will be dropped in course of the FEM estimation. Thus, although typically all constitutional variables of the above defined DiD-terms should be included in the regression specification of an interaction effect model (Brambor et al. 2006), the FEM setup, with the added country-pair fixed effects, makes this requirement obsolete (Angrist and Pischke 2010).

As shown in Eq. (2), we do not include time-varying covariates in the regression equation besides the time-constant country-pair fixed effects. The latter already capture all unobserved factors such as distance and common language, which do not vary over time. The inclusion of further time-varying control variables is controversially discussed in the literature and shows to have advantages and disadvantages (Lechner 2010). In our specific case of dyadic co-publication patterns it is hardly possible to find relevant covariates, which would help to control for potentially different time trends among the treatment and comparison group, while fulfilling the exogeneity assumption. That is, included time-varying covariates should not be affected by the outcome variable of interest in the post treatment period since they may lead to an estimation bias otherwise. The absence of covariates in the DiD-regression framework also has the advantage that no additional identification assumptions have to be stated.

## Results

Our results of the DiD-estimation setup according to Eq. (2), which include EU enlargement-related time trends for all six macro groups (as well as aggregates thereof), are summarized in Table 3. The models are estimated by means of pooled OLS including group dummies but excluding country-pair fixed effects (denoted POLS) as well as by means of FEM estimation including country-pair fixed effects. Thereby, the columns 1 and 2 only include the group-specific time trends for the first enlargement wave $\left( {\delta^{2004,g} } \right)$ and aggregate the two sub-populations of new EU members into one new member state group for the NMS-12. The column 3 then also include the additional group-specific time trends for the second enlargement wave $\left( {\delta^{2007,g} } \right)$ for the aggregated NMS-12 group. Finally, column 4 disaggregates the group-specific time trends for the five treatment groups $(T_{ij}^{2} \;{\text{to}}\;T_{ij}^{6} )$ and comparison group $(T_{ij}^{2} )$ as defined above.

**Table 3:** Estimation results for alternative DiD-model specifications

Group (g)	Collaboration among	Estimated coefficient	(1) POLS	(2) FEM	(3) FEM	(4) FEM
*EU enlargement 2004*						
*T*_*ij*_^1^	*EU*-*15/EU*-*15*	*δ* ^2004,*g*^	0.732*** (0.1327)	0.735*** (0.0999)	0.482*** (0.1468)	0.481*** (0.1461)
(*T*_*ij*_^2^ + *T*_*ij*_^3^)	*EU*-*15/NMS*-*12*	*δ* ^2004,*g*^	1.478*** (0.1670)	1.478*** (0.1257)	1.151*** (0.1847)	
*T*_*ij*_^2^	*EU*-*15/NMS*-*10*	*δ* ^2004,*g*^				1.319*** (0.1906)
*T*_*ij*_^3^	*EU*-*15/NMS*-*2*	*δ* ^2004,*g*^				0.310 (0.3100)
$\left( {T_{ij}^{4} + T_{ij}^{5} + T_{ij}^{6} } \right)$	*NMS*-*12/NMS*-*12*	*δ* ^2004,*g*^	3.963*** (0.2136)	2.963*** (0.1608)	2.446*** (0.2363)	
*T*_*ij*_^4^	*NMS*-*10/NMS*-*2*	*δ* ^2004,*g*^				1.154*** (0.3654)
*T*_*ij*_^5^	*NMS*-*10/NMS*-*10*	*δ* ^2004,*g*^				3.085*** (0.2668)
*T*_*ij*_^6^	*NMS*-*2/NMS*-*2*	*δ* ^2004,*g*^				−0.476 (1.5505)
*EU enlargement 2007*						
*T*_*ij*_^1^	*EU*-*15/EU*-*15*	*δ* ^2007,*g*^			0.381** (0.1621)	0.381** (0.1613)
$\left( {T_{ij}^{2} + T_{ij}^{3} } \right)$	*EU*-*15/NMS*-*12*	*δ* ^2007,*g*^			0.492** (0.2039)	
*T*_*ij*_^2^	*EU*-*15/NMS*-*10*	*δ* ^2007,*g*^				0.536** (0.2103)
*T*_*ij*_^3^	*EU*-*15/NMS*-*2*	*δ* ^2007,*g*^				0.270 (0.3423)
$\left( {T_{ij}^{4} + T_{ij}^{5} + T_{ij}^{6} } \right)$	*NMS*-*12/NMS*-*12*	*δ* ^2007,*g*^			0.777*** (0.2608)	
*T*_*ij*_^4^	*NMS*-*10/NMS*-*2*	*δ* ^2007,*g*^				1.497*** (0.4033)
*T*_*ij*_^5^	*NMS*-*10/NMS*-*10*	*δ* ^2007,*g*^				0.473 (0.2946)
*T*_*ij*_^6^	*NMS*-*2/NMS*-*2*	*δ* ^2007,*g*^				0.019 (1.6611)
	*T (Years)*		22	22	22	22
	*N (Country pairs)*		351	351	351	351
	*Within*-*R* ^2^			0.192	0.202	0.209

***, **, * denote statistical significance at the 1, 5 and 10 % critical level, respectively

Firstly, the results show an increasing trend in the co-publication intensity within the EU-15 (comparison group). What is marked is that (according to the regression specification in column 3 and column 4 of Table 3) both the first annexation period after 2004 as well as the period of 2007–12 were characterized by a positive growth trends of intra-EU-15 cross-border collaborations in terms of scientific articles, which grew on average by 48 % over the period 2004–12 with an additional growth impulse of 38 % throughout the sub-period 2007–12. This result signals that the trend line for growth has become steeper in the final parts (2004–6 and 2007–12) of the analyzed time period. In the following, the collaboration intensity within the EU-15 acts as the baseline against which the other treatments are benchmarked.

Secondly, with regard to the co-publication intensity between the old and new member states, the results in column 3 of Table 3 show that the scientific cross-border collaboration trend between these countries grew even stronger compared to the benchmark group of intra-EU-15 collaborations. On average, the co-publication intensity between the old and new member states $\left( {T_{ij}^{2} + T_{ij}^{3} } \right)$ grew by 115 % over the period 2004–12 with an additional growth stimulus of 49 % over the sub-period 2007–2012. When breaking the overall picture into two separate annexation groups (NMS-10 and NNS-2), it becomes evident from the estimation output in column 4 of Table 3 that this effect was mainly driven by an increase in the co-publication intensity of the EU-15 and NMS-10 (with an average growth rate of 132 % over the period 2004–12 and an additional growth rate of 54 % on top of the former for the sub-period 2007–12). However, for the NMS-2 the annexation of the NMS-10 in the first enlargement wave 2004 did not have any effect on their collaboration intensity between the established EU-15. Moreover, even in the period of the second enlargement wave starting from 2007, we do not observe a statistically significant positive growth trend for the collaboration intensity between the EU-15 and NMS-2.

Thirdly, the estimation results for the group-specific time trends in Table 3 further show that, throughout the two enlargement periods 2004 and 2007, we observe a strong acceleration in the collaboration intensity within the NMS-12, which strongly exceeds the growth rate for the comparison group of the EU-15. Specifically, according to column 3 of Table 3 the internal cross-border collaboration intensity of the NMS-12 grew by 245 % over the period 2004–12 and by additionally 78 % over the sub-period 2007–12. The strongest increase has been thereby experienced by the sub-group of NMS-10 (with an overall increase of 309 % in 2004–12). However, for this group we do not get evidence for an additional growth impulse over the sub-period 2007–12 indicating that the increase in the co-publication intensity has mostly taken place in the immediate aftermath of the EU accession of this group in 2004. Accordingly, the collaboration intensity between the NMS-10 and NMS-2 did witness an additional increase of 115 % when comparing the before–after 2004 co-publication intensity for this treatment group. This also holds for an additional acceleration in the co-publication intensity over the sub-period following the second EU enlargement wave of 2007. Here, the estimation results show an additional increase by 150 % in the co-publication intensity for this treatment group (column 4 of Table 3). However, no significant trend growth was detected within the final treatment group of NMS-2 for either enlargement periods.

Although the quantitative difference in the growth trends between the different groups becomes visible from the reported *δ*-coefficients in Table 3, we want to formally test for the existence of excess growth for the treatment groups *vis*-*à*-*vis* the comparison group for the time periods of EU enlargement. As the reported tests in Table 4 show, we find a positive and statistically significant excess long-run growth performance in the co-publication intensity across country pairs for three treatment groups NMS-10/NMS-2, NMS-10/NMS-10 and EU-15/NMS-10. Thereby, the statistics for the combined estimates according to Eqs. (3) and (4) indicate that the largest average treatment effect on the treated (ATT) in the observed co-publication intensity was achieved for the intra-NMS-10 group (270 %-points), followed by an increase in the co-publication intensity of the NMS-10 and NMS-2 (179 %-points) as well as an excess increase in the co-publication intensity of the EU-15 and NMS-10 (99 %-points). The NMS-2 only experienced an excess growth in their co-publication intensity with the NMS-10, while the growth in the co-publication intensity with the EU-15 did not exceed the benchmark growth rate of the intra-EU-15 co-publication intensity, but rather showed a relative decline (although not being statistically significant at the 10 % significance level).

**Table 4:** Ranking of estimated ATT effect of EU enlargement for different treatment groups

Group	Combined coefficient: $\delta_{ATT}^{g} = \left( {\delta^{2004,g} + \delta^{2007,g} } \right) - \left( {\delta^{2004,1} + \delta^{2007,1} } \right)$		Coef.	*t* test	*p* value
*T*_*ij*_^5^	NMS-10/NMS-10	Long-run ATT effect for 2004–12 (in %-points)	2.695***	9.51	(0.00)
*T*_*ij*_^4^	NMS-10/NMS-2	Long-run ATT effect for 2004–12 (in %-points)	1.788***	5.22	(0.00)
*T*_*ij*_^2^	EU-15/NMS-10	Long-run ATT effect for 2004–12 (in %-points)	0.992***	4.06	(0.00)
*T*_*ij*_^3^	EU-15/NMS-2	Long-run ATT effect for 2004–12 (in %-points)	−0.282	−0.91	(0.36)
*T*_*ij*_^6^	NMS-2/NMS-2	Long-run ATT effect for 2004–12 (in %-points)	−1.319	−1.12	(0.26)

***, **, * denote statistical significance at the 1, 5 and 10 % critical level, respectively. Underlying model coefficients are taken from column 4 in Table 3

**Fig. 3:**
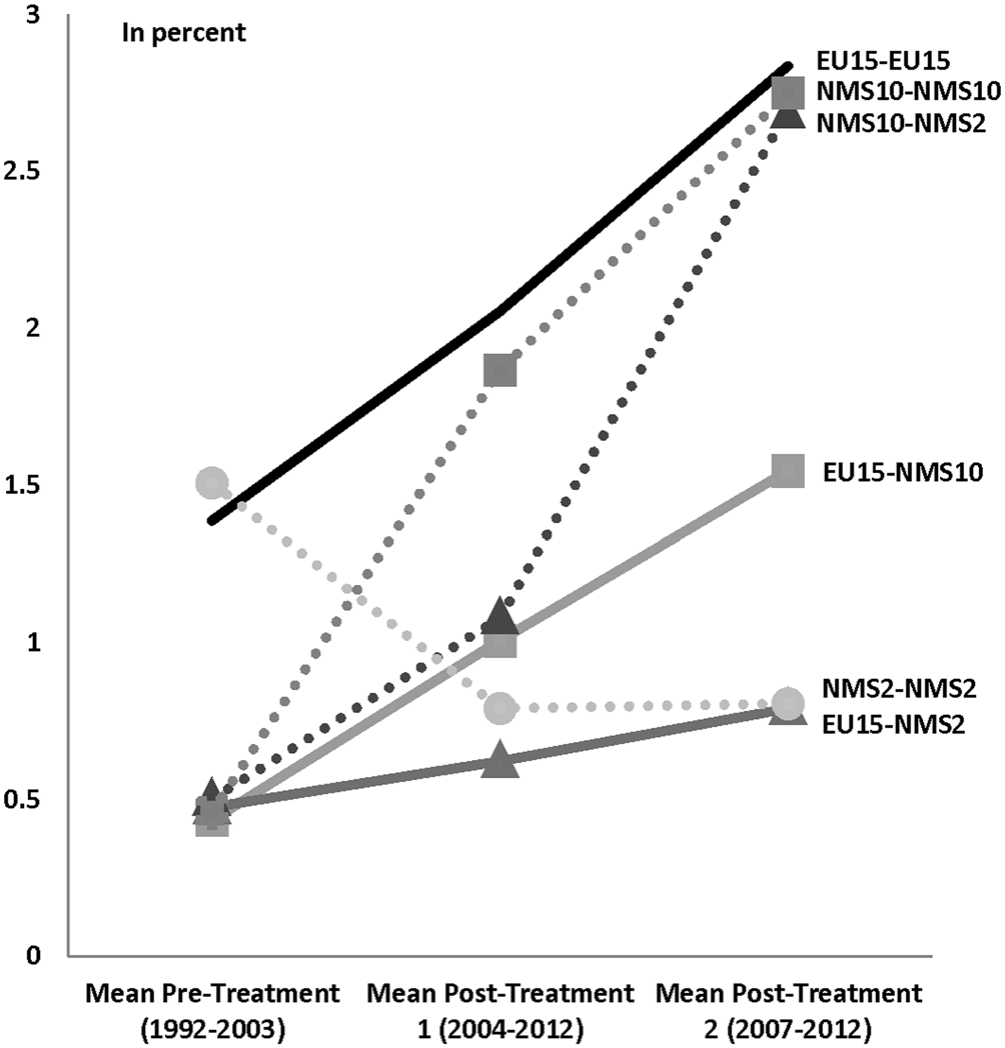
Predicted evolution in co-publication intensities for different country groups and treatment periods. *Note*: Predictions are based on regression coefficients from Table 3

In sum, the general picture obtained from Tables 3 and 4 hints at a statistically significant convergence tendency in the co-publication intensities towards the intra-EU-15 benchmark. This tendency is visualized in Fig. 3 based on the model predictions from column 4 in Table 3. As the Figure shows, by the end of the sample period both the co-publication intensity within the NMS-10 as well as scientific collaborations between the NMS-10 and NMS-2 have almost converged to the co-publication intensity within the EU-15 with average intensities between 2.69 and 2.84 %. The figure also shows that the co-publication intensity within the NMS-10 was mainly boosted throughout the first treatment period in 2004, while the co-publication intensity between the NMS-10 and NMS-2 picked up mainly afterwards. The picture for the co-publication intensity within the NMS-2 is quite different: the intensity started at a relatively high value in the pre-treatment period between 1992 and 2003 and then gradually declined. However, although these trends thus hint at convergence trends in the share of international publications, the gap in total numbers between the old and new member states is still large (as shown in Fig. 1 above).

## Robustness checks

**Fig. 4:**
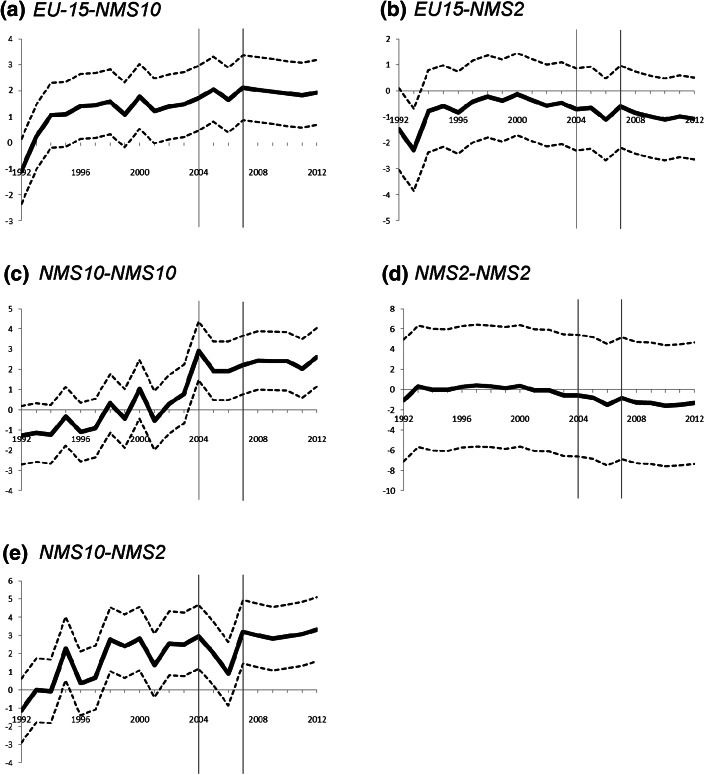
IDiD-coefficients for excess growth in the co-publication intensity of treatment groups; **a**–**e**. *Note*: The *solid line* show the estimated year-to-year IDiD-coefficients based on the combined estimates $\left( \delta_{\text{ATT}}^{{\text{t}},{\text{g}}} \right)$ for treatment groups (*g* = 2,…,6) using the delta method. *Dashed lines* indicate the *upper* and *lower* boundaries of the 95 % confidence interval

As outlined in the “Methods” section above, the common trend assumption is crucial for the reliability of the estimated DiD-parameters. Given that we have a panel data setup with several pre-treatment periods it is possible to test for the plausibility of this assumption (Lechner 2010). This is often done with the help of so-called ‘placebo’ experiments. The idea of placebo experiments is to pretend that the treatment has actually happened earlier and then measure the observed outcome difference after the ‘pretended’ treatment, but before the treatment actually happened. If the regression output then reports statistically significant effects, the reason for this could be twofold (Lechner 2010). Firstly, as already sketched above for the case of the NMS-2 throughout the first enlargement period 2004–2006, the treatment is anticipated and therefore has an effect even before it starts. This early anticipation is often also termed as ‘Ashenfelter’s Dip’ indicating a possible link between treatment and the idiosyncratic error term before treatment (Ashenfelter 1978). Secondly, if anticipation effects can be ruled out, any estimated non-zero effect has to be interpreted as selection bias and thus casts doubt on the validity of the identifying assumptions of the DiD-approach. Taking up this idea for a robustness check, we modify our regression specification as

5 $$y_{ij,t} = \mu_{ij} + \sum\limits_{i = 1992}^{I} {\sum\limits_{g = 1}^{G} {\delta^{t,g} \left( {D_{t}^{i} \times T_{ij}^{g} } \right)} } + \varepsilon_{ij,t} ,\quad \left( {for\;g = 1, \ldots ,6;\; i = 1992, \ldots , 2012} \right)$$

where we include individual time dummies for each sample year in the construction of the multiplicative DiD-interaction terms rather than the two multi-period dummies for the timing of the two enlargement waves 2004 and 2007. The resulting regression specification, also known as incremental Difference-in-Difference (IDiD) approach, allows capturing the average growth in the cross-border co-publication intensities for the six groups relative to the initial sample period 1991 (Dolton et al. 2010). One advantage of the IDiD-approach is that it facilitates the estimation of year-on-year incremental growth effects and can thus be used for the computation of placebo experiments. As before, we are primarily interested in obtaining parameter estimates for excess growth of treatment groups compared to the benchmark group along the line of the combined coefficient test outlined in Eqs. (3) and (4). When applying the IDiD-approach one has to note, though, that one cannot deduce the longer-run effect of the outcome changes in the course of treatment, as shown in Eq. (4), by simply summing up all the year-to-year IDiD-coefficients (Dolton et al. 2010). This is due to the fact that some additional (untestable) assumption regarding related to the interdependence of the obtained effects would be required. However, the approach still allows us to see whether there are early anticipation effects or not. The resulting IDiD-coefficients for the five treatment groups (*g* = 2,…,6), which are defined as net growth difference relative to the comparison group as $\delta_{ATT}^{t,g} = \left( {\delta^{t,g} - \delta^{t,1} } \right)$, are plotted in Fig. 4 together with a 95 % level confidence interval.

As Fig. 4 shows, particularly for the co-publication intensity between the EU-15 and NMS-10, we observe an excess growth *vis*-*à*-*vis* the growth trend within the EU-15, which started ahead of the first EU enlargement wave in 2004. This hints at the presence of some early anticipation effects in the aftermath of the political, social and economic transformation of the Eastern and Central European countries. As graph (a) in Fig. 4 indicates, the boost in the co-publication intensity between the old (EU-15) and new (NMS-10) member states became apparent in the middle of the 1990s rather shortly after the ‘fall of the iron curtain’ and the excess growth continued to be positive throughout the remainder of the years in the sample period. A similar pattern, albeit with a higher volatility in the reported year-to-year effects, can be observed for scientific collaborations between the NMS-10 and NMS-2 in graph (e) of Fig. 4.

While we do not find significantly positive year-to-year effects for the co-publication intensity within the NMS-2 (quite possibly also partly due to the modest numbers of co-publications between Bulgaria and Romania), nor in the case of the co-publication intensity between the EU-15 and NMS-2 (in fact, the visualizations—graphs (b) and (d)—in Fig. 4 rather hint at a decline), the excess growth in the co-publication intensity within the NMS-10 (graph (c) in Fig. 4) is shown to have the ‘right timing’ without any early anticipation effects. That is, only starting in 2004 the year-to-year effects for scientific collaborations between these countries can be shown to (statistically significantly) outperform the yearly growth effects in the benchmark group. Accordingly, whereas the co-publication trend between the NMS-10 and NMS-2 (graph (e) of Fig. 4) declined after the first wave of integration they quickly stabilized to their earlier level after the second wave of integration. These results support the general picture drawn from Tables 3 and 4. They also hint at the existence of positive integration and outcome effects of EU enlargement, most visibly for the co-publication intensity within the NMS-10.

## Discussion

The results clearly show that the most significant impact, in terms of co-publication intensities, of the EU enlargement has been the high increase in the level of scientific collaboration that the NMS-12 have among each other (this applies in particular to the NMS-10). Additionally, the collaboration between the new and old member states has been affected by the EU enlargement waves 2004 and 2007. Thus, the hypothesized impacts of the EU membership in boosting the research collaboration of the new member states (Marshakova-Shaikevich 2006, 2007) are generally confirmed. The results thus give tentative evidence supporting the success of the EU in achieving a common internal market in research. Whether or not this signals the success of the ERA and/or a particular strategy or a policy of the EU, however, remains outside the scope of this study. Rather, we can conclude that an EU membership status significantly increases the collaboration between a specific new member state and the other EU member states.

In short, the increase in the collaborations between the NMS-12 and EU-15 started immediately after the dissolution of the Soviet Union in the mid-1990s, but joining the EU has had an additional positive long-run impact on the international scientific collaboration intensity of the NMS-12 (NMS-10 in particular) in terms of the rising numbers of co-publications between themselves and the established EU-15. While early anticipation effects are particularly present for the cross-border collaborations between the EU-15 and NMS-10 (most likely due to the ‘pull effect’ of the established research market in the EU-15), especially the excess growth within the NMS-10 scientific collaborations are shown to have the right timing underlying the existence of causal effects of EU enlargement on the cross-border co-publication intensity.

**Fig. 5:**
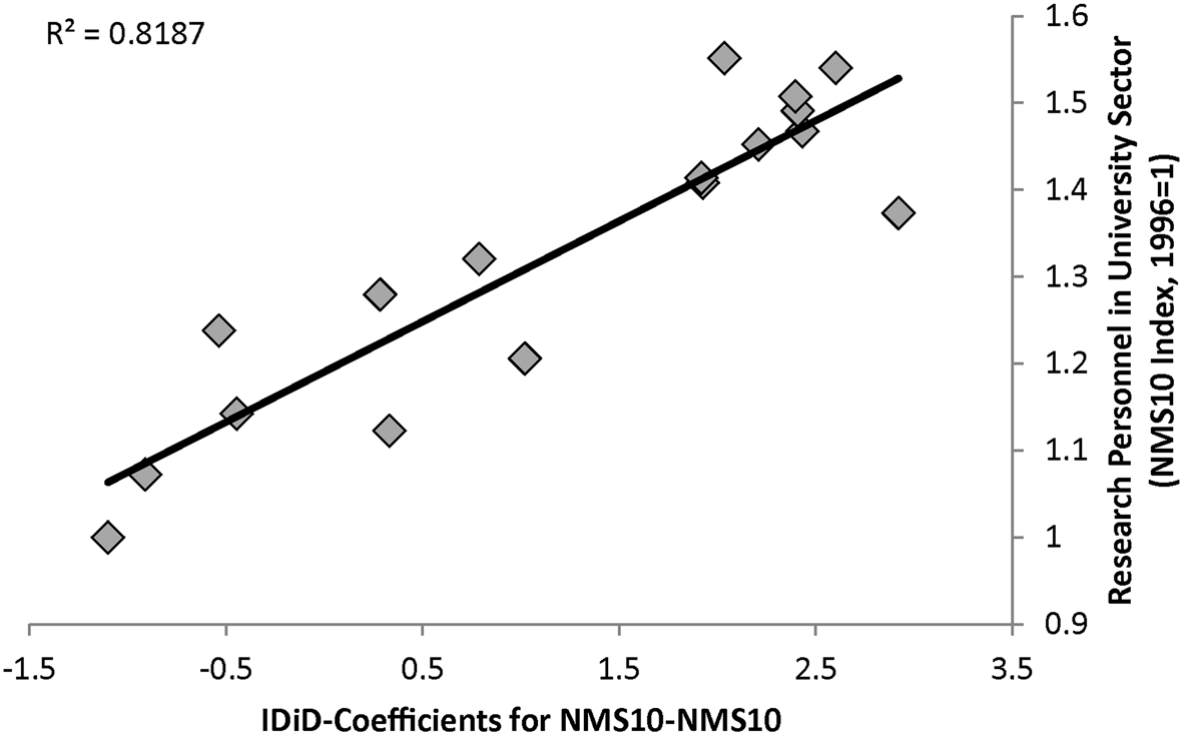
Correlation between IDiD-coefficients and research personnel in the NMS-10 (1996–2012). *Source* Own calculations based on data from Eurostat (2014). *Note* Research personnel defined as researchers in the university sector for NMS-10 aggregate. Predictions are based on displayed regression coefficients from graph c in Fig. 4. Index for research personnel in the university sector calculated for base year 1996 = 1

This latter result, especially, can be seen as a valuable input for a policy-oriented discussion. Obviously, the change in the institutional setup as achieved via EU accession is a necessary but not sufficient condition to foster European co-publication intensity of its member states. Thus, what is further needed is a complementary focus on the absorptive capacities in lagging regions. For instance, if we correlate the estimated yearly IDiD-coefficients from Fig. 4 for the co-publication intensity within the NMS-10 with an index for the evolution of research personnel in the university sector for the NMS-10 country aggregate, we can gather from Fig. 5 that the non-linear pattern of EU enlargement effects, as estimated by the IDiD-approach, is highly correlated (with a correlation coefficient of *R*^2^ = 0.82) with a similar dynamic increase in the number of research personnel linked to the EU accession of these countries and their integration into the ERA.

Contrary to the observations for the NMS-10, the integration of Bulgaria and Romania into the scientific system of the EU can only partially be observed. Here especially an excess growth in the co-publication intensity with the NMS-10 can be observed. The latter boost in the scientific collaboration intensity has thereby already started before these two countries actually became EU member states. Thus, it seems that even the anticipation of subsequent EU enlargement can have a positive impact on the collaboration intensity between contemporary EU and non-EU countries. This integration, however, has neither happened within the NMS-2 themselves nor between these countries and the EU-15. Potential reasons for this partial non-integration of Bulgaria and Romania may be that not enough time has passed yet for these countries to break into the European networks of scientific collaboration, arguably due to their ‘weaker’ similarity to the West than in the case of the NMS-10 (Mirskaya 1997; Must 2010). Additionally, the earlier EU accession of the NMS-10 may have resulted in the creation of stable collaboration networks between the EU-15 and NMS-10, which may hinder the NMS-2 to enter the internal EU research market.

Based on our results the process of internationalization (or at least Europeanization) of science seems to be far from reaching its end, contrarily to what has been suggested by Ponds (2009), since the sheer numbers of collaborations have continued to grow throughout the time period analyzed with an increasing ‘velocity’ (Fig. 1). Additionally, whereas in 1991 for every 100 articles there were eight international collaboration partners from different EU countries, the corresponding figure has risen steadily to thirty-five in 2012 (this ratio has risen also individually per every analyzed EU country). Particularly, it seems that the new member states are catching-up to the established ones in terms of the share and total numbers of intra-EU co-publications. At the moment, however, the gap between them can be described as relatively large and the velocity of the convergence process as fairly modest.

The presented results have definite policy implications. As we have observed significant early-anticipation effects of consecutive EU accession, it appears to be important to coordinate science and technology policies between the established EU countries and upcoming new members already prior to their entry in order to guarantee a smooth transition process into the ERA for the research institutions of the new entrants. In line, it seems that the scientific networks inside the EU are quite ‘oligarchic’: in fact, the impact of EU accession on cross-border co-publication was found to be more significant within the NMS-12 rather than between this group and the established EU-15. Furthermore, the importance of public spending related to research inputs (proxied by research personnel in the university sector in Fig. 5) has been highlighted here as an important driver of cross-border co-publication patterns and integration. Therefore, for EU-wide cohesion to take place, it would be beneficial to secure a certain level of research funding targeted specifically to recipients other than the leading public and private research actors in the EU-15, for example through separate funding instruments, in order to allow the new member states to build up a stock of research competence and infrastructure. This would enable them to catch-up with the established EU-15 institutions. Alternatively, a gradual opening of highly restricted network structures in the field of research and development may also be achieved by incentivizing leading research actors in the EU-15 to round up partners outside their list of ‘usual suspects’ by supporting them in collaborating with and transferring knowledge to emerging actors in the new member states.

The structure of the EU’s new ‘Horizon 2020’ programme to support excellence in research and innovation can be seen as one step into this direction. Particularly the newly introduced scheme ‘Spreading excellence and widening participation’ within the ‘Horizon 2020’ programme addresses the mismatches of research and innovation activities between the old and new member states and offers various measures for overcoming these existing imbalances (European Commission 2015). As such, these measures are mainly targeted to provide support for universities and other research institutions located in low-performing member states for establishing new scientific networks (‘teaming’) and partnerships with internationally-leading counterparts in Europe (‘twinning’).

## Concluding remarks

At the end, it has to be noted that our chosen DiD-approach applied to the WoS data does contain some limitations (as discussed in the “Data and methods” section). In line, the levels of integration in co-publishing are likely to vary according to different disciplines and research fields (Luukkonen and Nedeva 2010). Additionally, it has to be acknowledged that other factors such as the national scientific infrastructure, trade flows, cultural ties and research funding mechanisms can play significant roles in supporting and sustaining the publication patterns in cross-border collaboration (Libkind 2014; Ukrainski et al. 2014; Cassi et al. 2015). For example, one could take into account the opportunities for participation in EU funded research projects as a mediating factor, which provide the rationale for joint publications later on. Thus, integrating panel data on the scientific infrastructures, previous levels of research, specific research fields, joint projects funded by the EU and cross-country trade flows as well as applying extended econometric models for analyzing causal linkages would be interesting directions for further analysis. However, acquiring such data for the time period analyzed here and applying causality tests—other than the DiD-approach—are not without their own difficulties and limitations. Still, our seemingly simple empirical approach was able to pinpoint several interesting results to be further discussed and tested in subsequent studies:

- First, the number of co-publications between the old and new member states started to grow immediately after the dissolution of the Soviet Union.
- Second, an EU membership status significantly increases the collaboration between a specific new member state and the other EU countries.
- Third, early anticipation effects of consecutive EU accessions are also clearly visible i.e. the anticipation of subsequent EU enlargement has a positive impact on the collaboration intensity between present and future EU countries.
- Fourth, the process of internationalization in scientific collaborations seems to be far from reaching its end.
- Fifth, there is a convergence tendency between the cross-border co-publication intensities of the old and new member states.
- Sixth, the results support (tentatively) the views underlining the positive integration effects of EU’s science and technology policies.

## Footnotes

1 Austria, Belgium, Denmark, Finland, France, Germany, Greece, Ireland, Italy, Luxembourg, Netherlands, Portugal, Spain, Sweden and United Kingdom.

2 Bulgaria, Cyprus, Czech Republic, Estonia, Hungary, Latvia, Lithuania, Malta, Poland, Slovakia, Slovenia and Romania.
